# Navigating the New Era in Myelodysplastic Neoplasms: A Review of Prognostic Implications of the IPSS-M Score and 2022 WHO Classification

**DOI:** 10.3390/hematolrep17060058

**Published:** 2025-10-30

**Authors:** Mihai-Emilian Lapadat, Oana Stanca, Nicoleta Mariana Berbec, Cristina Negotei, Andrei Colita

**Affiliations:** 1 Department of Hematology, “Carol Davila” University of Medicine and Pharmacy, 050474 Bucharest, Romania; oana.stanca@umfcd.ro (O.S.); nicoleta.berbec@umfcd.ro (N.M.B.); cristina.negotei@umfcd.ro (C.N.); andrei.colita@umfcd.ro (A.C.); 2 Clinic of Hematology, Coltea Clinical Hospital, 030171 Bucharest, Romania

**Keywords:** myelodysplastic neoplasms, myelodysplastic syndromes, WHO 2022 classification, IPPS-M, risk stratification, prognostic models, cytogenetics

## Abstract

Myelodysplastic neoplasms represent a diverse group of clonal hematopoietic stem cell disorders characterized by ineffective hematopoiesis, peripheral cytopenias, and an inherent risk of progression to acute myeloid leukemia. Accurate risk assessment and patient stratification are critical to optimizing therapeutic approaches and clinical outcomes. In 2022, significant advancements reshaped both the classification and prognostic stratification of MDSs. The revised WHO Classification introduced crucial genetically defined subtypes, particularly those involving biallelic *TP53* inactivation and *SF3B1* mutations, shifting the emphasis from traditional morphology-based criteria to molecular ones. Simultaneously, morphological subtypes such as hypoplastic and hyperfibrotic MDSs were established as distinct entities with unique prognostic implications. At the same time, the introduction of the International Molecular Prognostic Scoring System (IPSS-M) provided a more precise prognostic stratification by integrating comprehensive molecular data alongside traditional clinical and cytogenetic parameters. Several validation studies have confirmed IPSS-M’s superior discriminative power compared to previous models, notably IPSS-R, improving predictions regarding overall survival and leukemia transformation. Nevertheless, practical considerations regarding the widespread application of IPSS-M have emerged, including concerns over economic feasibility and accessibility of advanced molecular testing methods, such as extensive Next-Generation Sequencing panels. This review synthesizes the recent literature and critical studies validating these classification and prognostic updates, discussing their clinical impact, practical considerations, and implications for targeted therapeutic strategies. By focusing on molecular pathogenesis, the latest classification systems and prognostic models promise significant advances in patient-specific management, setting the stage for future innovations in treatment and improved patient outcomes.

## 1. Introduction

Myelodysplastic neoplasms (MDSs) represent a heterogeneous group of clonal hematopoietic stem cell disorders characterized by the progressive accumulation of genetic alterations, which impairs effective cell formations [1]. These changes in the hematopoietic landscape lead to inefficient hematopoiesis manifesting clinically as varying degrees of peripheral cytopenias and are accompanied by characteristic morphological abnormalities [2]. These morphological abnormalities—defined by dysplasia in one or more cell lineages—are a hallmark of the disease and are often associated with increased cellular apoptosis. In addition, MDSs are marked by highly variable cytogenetic profiles [3] and carry an intrinsic risk of progression to acute myeloid leukemia (AML) [4].

MDSs rank fifth among malignant hematological disorders in terms of incidence. The reported overall incidence ranges for 2 to 5 cases per 100,000 individuals [5,6], with a median age at diagnosis of approximately 71 years [7]. However, more recent population-based registries suggest a rising median age at onset, now approaching 74–75 years [8]. A slight male predominance has been consistently observed, with a sex ratio of approximately 55% male to 45% female [6]. Incidence rises sharply with advancing age, surpassing 50 cases per 100,000 in individuals over 80 years old [9].

Although the strength of evidence varies across exposures, several risk factors for myelodysplastic neoplasms (MDSs) are consistently discussed in the literature. As noted above, older age and male sex show a clear epidemiologic association with MDSs [3]. Prior cytotoxic chemotherapy and/or radiotherapy represent the most firmly established risks, supported by numerous cohort and registry studies [10,11]; accordingly, contemporary classifications recognize a distinct category of myeloid neoplasms “with prior cytotoxic therapy” [2]. Occupational or environmental benzene exposure is another well-documented risk factor, with strong links to MDSs and other hematologic malignancies given benzene’s capacity to induce leukemia-associated cytogenetic alterations [12,13]. Additional exposures—such as pesticides or organic solvents [14]—and inflammatory factors (with smoking being the most consistently reported [15]) have been variably associated across studies, but the aggregate evidence is less robust than for prior cytotoxic therapy or benzene.

## 2. Historical Overview of MDSs’ Classification

The first mention of what is now known as myelodysplastic neoplasm appeared in the literature in 1953, under the term “preleukemia.” Over the following decades, several alternative designations were used, including “acute smoldering leukemia,” “preleukemic syndrome,” and “subacute myeloid leukemia.” The term “myelodysplastic syndrome” was formally introduced in 1982, coinciding with the first standardized classification system—the French-American-British (FAB) classification. It recognized five distinct entities: refractory anemia (RA), refractory anemia with ringed sideroblasts (RARS), refractory anemia with excess blasts (RAEB), refractory anemia with excess blasts in transformation (RAEB-T), and chronic myelomonocytic leukemia (CMML) [16].

In 2008, the World Health Organization (WHO) introduced its own classification system for MDS, refining diagnostic criteria and incorporating new and pathological insights [17]. Subsequently, the classification of myelodysplastic neoplasms was revised by the World Health Organization in 2016, resulting in more precisely defined subtypes: myelodysplastic syndrome with unilinear dysplasia, with multilinear dysplasia, with ring sideroblasts and unilinear dysplasia, with ring sideroblasts and multilinear dysplasia, with del(5q), with excess blasts type I, with excess blasts type II and unclassifiable [18].

In 2022, WHO released a revised classification of MDS (now officially termed *myelodysplastic neoplasms*) [19], introducing a major shift by separating MDS into two broader categories: genetically defined and morphologically defined myelodysplastic syndromes. Within the genetically defined group, MDS with isolated del(5q) was retained from earlier classifications, while two newly recognized entities were added: MDS with biallelic *TP53* inactivation, which superimposes over several prior subtypes, and MDS with *SF3B1* mutation, which overlaps with the former category of MDS with ring sideroblasts, as well as partially with unilineage and multilineage dysplasia subtypes. As for the morphologically defined category, the central element remains the percentage of blasts in the bone marrow and peripheral blood. This group includes MDS with low percentage of blasts, which now includes hypoplastic MDS and MDS with low blast percentage (“low blast”), and MDS with high percentage of blasts, which now comprises subtypes with increased blasts type 1, increased blasts type 2 and MDS with associated bone marrow fibrosis. A detailed, side-by-side comparison of the 2016 and 2022 WHO Classifications of MDS–including the key changes and redefined entities–is presented in Table 1.

Additionally, in 2022, the International Consensus Classification (ICC) for Myeloid Neoplasms and Acute Leukemias introduced an updated framework for myelodysplastic neoplasms that further foregrounds molecular and cytogenetic features (e.g., *SF3B1* mutation, isolated del(5q)) [20]. Notably, the ICC defined a new transitional category, “MDS/AML,” encompassing cases with 10–19% bone marrow blasts, thereby replacing the former “excess blasts-2” designation and explicitly signaling proximity to acute myeloid leukemia. The revised nomenclature emphasizes the clinical importance of medullary blasts by using the 10% threshold as a gateway toward AML biology and management. This approach partially overlaps with WHO 2022, which retains such cases within MDS with increased blasts-2 (MDS-IB2) but acknowledges that certain AML-defining genetic abnormalities permit an AML diagnosis irrespective of a fixed blast percentage. Together, the two systems converge on a more genetics-informed taxonomy while sharpening attention to blast percentage as a critical marker of disease trajectory. A direct comparison of the WHO 2022 and ICC 2022 Classifications can be found in Table 2. 

## 3. Prognostic Scoring Systems in MDSs

Risk stratification plays a central role in both estimating prognosis and therapeutic decision-making, guiding the selection of patients to benefit from supportive care versus disease-modifying interventions. The first widely adopted prognostic tool was the International Prognostic Score (IPSS [21]), introduced in 1997. This model integrated three key parameters—bone marrow blast percentage, cytogenetic abnormalities, and the number of peripheral cytopenias—to stratify patients into four risk categories: low, intermediate-1, intermediate-2, and high. 

In 2010, the World Health Organization Prognostic Scoring System (WPSS) was developed [22], incorporating WHO morphologic subtype, cytogenetics, and transfusion dependence to produce five risk groups: very low, low, intermediate, high, and very high. 

A further refinement came with the Revised IPSS (IPSS-R, 2012 [7]), offering an expanded cytogenetic risk classification and incorporating not only the type, but also the depth of cytopenias resulting in the same five-tier structure as WPSS: very low, low, intermediate, high, and very high. 

In addition to these major systems, several alternative models have been proposed—such as the M.D. Anderson Lower-Risk Prognostic Scoring System [23], the Global MD Anderson Model [24], and the French Prognostic Scoring System [25]—though these are of lesser importance and used less frequently in routine clinical practice. 

In 2022, the International Prognostic Working Group on Myelodysplastic Syndromes (IWG-PM) introduced the International Molecular Prognostic Score (IPSS-M) [26]. This model was derived from a cohort of 2957 untreated, newly diagnosed MDS patients and externally validated in an independent cohort of 754 patients. Building on the clinical and cytogenetic variables of the IPSS-R, the IPSS-M incorporates (1) patient-related clinical features—age, depth of cytopenias, and bone marrow blast percentage—and (2) cytogenetic risk categories using the same stratification as IPSS-R, but it also adds the crucial concept of (3) molecular genetics, evaluating 31 recurrent gene mutations leading to a more accurate risk stratification and prognosis. The model stratifies patients into six prognostic groups: very low, low, moderately low, moderately high, high, and very high. A comparison of IPSS, IPSS-R and IPSS-M can be found in Table 3.

## 4. Clinical Validation and Impact of the 2022 WHO Classification

The year 2022 marked a pivotal moment in the field of MDSs, which benefited from substantial revisions to both their classification and prognostic frameworks, which ultimately leads to an increased benefit in terms of the patient and their therapeutic approach. These developments have had a direct and meaningful impact on patient management, enabling more refined therapeutic strategies and ultimately enhancing clinical outcomes.

As previously discussed, 2022 marked the publication of the fifth edition of the *WHO Classification of Tumours of the Haematopoietic and Lymphoid Tissue* [13]. A key update of this classification concerns terminology: while the familiar term and concept of “syndrome” are retained (along with the “MDS” abbreviation), the condition is now referred to as myelodysplastic neoplasm. This change was made in an effort to both emphasize the neoplastic nature of the disease and to harmonize its nomenclature with that of myeloproliferative neoplasms.

From a morphological standpoint, the stratification of patients based on blast percentage remains a valid criterion, distinguishing between low- and high-blast categories to guide risk assessment and prognostic evaluation. Notably, the revised classification delineates hypoplastic myelodysplastic syndrome and hyperfibrotic syndrome as distinct entities, reinforcing the necessity of bone marrow biopsy at diagnosis. The inclusion of these subtypes further reflects a shift in diagnostic emphasis, suggesting that the concept of “excess blasts” is losing its importance and now serves primarily as a diagnostic criterion when other defining morphological and molecular features are absent. In the absence of these features, the presence of blasts remains an important criterion as shown by ICC 2022 who suggests that these patients should be considered and treated as AML.

The updated classification also places greater emphasis on genetically defined myelodysplastic neoplasms, introducing two new subtypes alongside the already established MDS with isolated del(5q). This refinement reflects significant progress in understanding the underlying pathogenic mechanisms—an advancement of particular importance given the therapeutic implications of a deeper molecular understanding of the disease. 

Preliminary data we have since the introduction of the new classification appear to support and validate its superior discriminative power. In one such study [27], 852 patients originally stratified according to the 2016 criteria were re-classified under the updated framework, which underscored the significant role of genetic mutations in refining risk assessment. 

Consequently, thirty patients found to carry an *NPM1* mutation were re-classified as having AML with *NPM1* mutation, given that under the updated classification the presence of this mutation is sufficient for a diagnosis of acute leukemia, irrespective of bone marrow blast percentage. Notably, these patients had previously been categorized under various myelodysplastic subtypes, including MDS with excess blasts type 1 and 2, multilineage dysplasia, and unclassifiable MDS. This reclassification highlights how the updated stratification consolidates genetically driven cases under a more precise—and clinically actionable—diagnostic category, while preserving and more accurately reflecting the underlying narrative of disease biology. 

The introduction of the new subtype defined by the presence of an *SF3B1* mutation led to the reclassification of 25 patients who had previously been categorized as having MDS without excess blasts. This shift comes from the strong association between *SF3B1* mutations and the former MDS subtype with ring sideroblasts [28]. Unlike the previous classification, which required ≥15% ring sideroblasts, the updated subtype is defined primarily by the presence of the *SF3B1* mutation, allowing for the inclusion of patients who would not have met the prior morphologic threshold.

Recent studies [28,29] have demonstrated both the significant correlation between *SF3B1* mutations and myelodysplastic neoplasms and a relative prognostic homogeneity among mutation-positive cases. As a result, the new classification replaces the former ring sideroblasts subtype with the genetically defined *SF3B1*-mutant subtype, which also partially overlaps with the former unilineage and multilineage dysplasia subtypes. This refinement carries meaningful therapeutic implications, particularly by broadening the population of patients eligible for targeted therapies—such as Luspatercept, which was initially indicated for patients with ring sideroblasts at the time of the updated classification, but has since received expanded approval for use in all lower-risk MDS [30,31].

Another significant update is the definition of a new subtype of myelodysplastic neoplasm with biallelic *TP53* inactivation, resulting in the reclassification of 53 patients—most of them previously defined as myelodysplastic syndrome with excess blasts (34 out of 53 patients). Additionally, 42 patients initially identified as having excess blasts were reclassified into the newly defined subtype of MDS with bone marrow fibrosis. The clinical significance of establishing these new entities is highlighted by distinct differences in median survival: 10 months for patients with MDS with biallelic *TP53* inactivation, and 15 months for those with bone marrow fibrosis, compared to 45 months for the entire study cohort. 

The unfavourable prognosis associated with these newly defined entities is further supported by the increased median survival observed among patients with increased blasts after excluding individuals reclassified into the two new categories from survival analysis. Specifically, median survival improved to 24 months for patients with increased blasts-1 and 26 months for those with increased blasts-2 after removing patients redefined as MDS-biTP53 and MDS-fibrosis, compared to 23 months for patients with excess blasts type 1 and 17 months for those with excess blasts type 2 according to the previous 2016 classification. Both the similar median survival between increased blasts-1 and -2, and the notable differences compared to the *TP53*-biallelic inactivation and fibrosis subtypes, emphasize that bone marrow blast percentage alone is no longer a decisive prognostic marker. Instead, these findings reinforce the necessity of integrating additional morphological and molecular markers to more accurately evaluate prognosis and guide therapeutic strategies in patients with MDS. 

## 5. Clinical Validation and Impact of IPSS-M

In addition to the prognostic refinements resulting from the updated classification of patients with MDS, there has also been significant improvement in prognostic stratification through the introduction of the new molecular scoring system, IPSS-M. Multiple studies have already validated this prognostic model across various patient cohorts, and several have further demonstrated its superior accuracy compared to previously established scoring systems.

The original study leading to the development of the IPSS-M score provided several important insights [26]:

(1) Genetic mutations were identified in 90% of the study participants, whereas cytogenetic analysis revealed chromosomal abnormalities in only 41% of patients, emphasizing the essential role of comprehensive molecular testing.
(2) Three mutations emerged as indicators of an especially poor prognosis: *TP53* “multi-hit”, *FLT3*, and *MLL-PTD*. In addition, *FLT3* and *MLL-PTD* mutations demonstrated a strong association with an increased risk of leukemic transformation.
(3) Mutations involving *ASXL1*, *BCOR*, *EZH2*, *NRAS*, *RUNX1*, *STAG2*, and *U2AF1* correlated significantly with adverse outcomes across the study’s primary endpoints: overall survival, leukemia-free survival, and progression to acute leukemia.
(4) Mutations in SF3B1 were associated with a generally favorable prognosis, although the outcomes varied depending on additional genetic alterations. The most favorable subgroup, labeled SF3B1β, comprised patients with *SF3B1* mutations combined with any mutation other than those defining the SF3B15q subgroup (*SF3B1* mutation plus del(5q)) or the SF3B1α subgroup (*SF3B1* mutation plus concurrent mutations in *BCOR*, *BCORL1*, *NRAS*, *RUNX1*, *SRSF2*, or *STAG2*).
(5) The study resulted in the reclassification of 46% of patients compared to their original IPSS-R classification. Within this group, 74% moved to a higher-risk category, whereas 26% shifted to a lower-risk category. A notable finding was that among patients originally stratified as intermediate-risk by IPSS-R, 21% were reclassified into the very high-risk IPSS-M category.
(6) Finally, myelodysplastic neoplasms with prior cytotoxic therapy exhibited a higher prevalence of complex karyotypes and monosomy 7. Nevertheless, 39% of these patients were re-stratified into the very low or low-risk categories according to IPSS-M.

Firstly, we observe a tendency to overlap the morphological classification and IPSS-M score. Specifically, the IPSS-M score independently identified the biallelic *TP53* mutation and the *SF3B1* mutation as having prognostic impact, confirming to some extent the superior efficiency of the new classification and the importance of molecular prognostic factors. 

The study emphasizes once again the importance of deep molecular investigations: approximately 50% of patients had normal karyotypes by cytogenetic analysis, yet bore clinically relevant molecular mutations, some of which influence prognosis in terms of survival and risk of leukemic transformation. 

Finally, IPSS-M proved to be superior compared to IPSS-R in terms of risk stratification: approximately 37% of patients were up-staged into higher-risk categories. This superiority was further validated by an improved concordance index in terms of leukemia transformation-free survival, overall survival, and risk of leukemic transformation.

Subsequent studies have validated the IPSS-M score in diverse patient populations [32,33,34,35], although most of these were retrospective and relied primarily on initial Next Generation Sequencing (NGS) data collected at diagnosis or prior to initiating therapy, without fully accounting for the potential of clonal evolution in MDS.

In one particular study, Sabile et.al [32] validated the IPSS-M in a cohort of 149 patients in which 32% of patients were up-staged and 12% down-staged compared to their initial IPSS-R stratification. IPSS-M demonstrated superior discriminative capability over IPSS-R, as evidenced by higher concordance index values for leukemia-free survival (0.72 vs. 0.68) and overall survival (0.74 vs. 0.70). 

Similarly, Baer et al. [33] also confirmed the superiority of IPSS-M in this regard. Approximately 25% of the patients were up-staged according to IPSS-M, correlating with improved predictive performance reflected by higher c-index values: 0.71 vs. 0.68 for overall survival, 0.73 vs. 0.69 for leukemia-free survival, and 0.81 vs. 0.77 for leukemic transformation. 

In another study, Sauta et al. [34] evaluated IPSS-M in two patient groups: those undergoing allogeneic hematopoietic stem cell transplantation (HSCT) and those receiving hypomethylating agents (HMAs). Among transplanted patients, IPSS-M offered improved predictive accuracy for post-transplant relapse and survival compared to IPSS-R, even after adjusting for cofactors such as age, sex, interval from diagnosis to transplantation, disease status at transplantation, and conditioning regimen. However, a notable limitation was the reliance solely on diagnostic data without accounting for treatments or repeat pre-transplant NGS assessments, potentially impacting statistical precision. Conversely, no statistically significant differences in treatment response according to IPSS-M risk categories were observed among patients receiving HMAs.

Lastly, Aguirre et al. [35] validated IPSS-M in patients with prior cytotoxic therapy myelodysplastic neoplasms, finding nearly half of the cohort reclassified into different risk categories. Survival without leukemic transformation was significantly dependent on the IPSS-M risk classification. Importantly, approximately 10% of patients previously categorized as low or intermediate risk by IPSS-R were re-stratified into a high-risk category by IPSS-M.

However, it should be noted that there are studies that although validated the superiority of the IPSS-M score, questioned the practical usefulness of this score. One analysis [36] indicated that while around 43% of patients were restratified using the IPSS-M score, only 17% were considered therapeutically relevant—patients who from a lower risk IPSS-R category were up-staged to a higher risk IPSS-M category. Moreover, after taking into consideration clinical characteristics such as age and comorbidities alongside institutional standard of care, only 9.5% of patients would have received different treatment if stratified according to the IPSS-M score, suggesting limited practical applicability.

The necessity for prospective studies is of outmost importance, particularly to identify high-risk patients who might benefit from interventional therapies such as hypomethylating agents or allogeneic hematopoietic stem cell transplantation. In this context, we believe borderline-risk patients—those who do not clearly fall into low- or high-risk groups by IPSS and/or IPSS-R—may derive particular value from IPSS-M. Because IPSS-M incorporates molecular data, applying it to these indeterminate cases may sharpen risk stratification and more reliably pinpoint candidates for therapeutic intervention.

Finally, considering the complexity, cost, and limited accessibility of a comprehensive 31-gene NGS panel, the overall clinical and economic benefits of broadly applying IPSS-M-driven reclassification warrant further discussion and consideration.

## 6. Conclusions

Recent significant advancements in the classification and prognosis of myelodysplastic syndromes have brought about a new era of risk stratification, ultimately enhancing patient outcomes and therapeutic decision-making.

Key changes include reduced emphasis on bone marrow blast percentage in defining MDS subtypes, alongside the introduction of two genetically defined entities characterized by *SF3B1* mutations (replacing the previous category of MDSs with ring sideroblasts and partially merging unilinear and multilinear dysplasia subtypes) and biallelic *TP53* mutations. Additionally, the updated classification recognizes MDSs with marrow fibrosis and MDSs with hypoplasia as distinct entities, each with unique clinical courses and prognoses.

Regarding the IPSS-M molecular Prognostic Scoring System, existing research predominantly validates its effectiveness across various patient cohorts, demonstrating that nearly half of patients undergo reclassification compared to the IPSS-R. However, as most studies have been retrospective and relied on initial diagnostic data, further prospective investigations are required to evaluate the IPSS-M’s predictive accuracy in the context of MDS clonal evolution and to determine its cost-effectiveness, given the resource-intensive nature of comprehensive genetic testing. This is especially pertinent for borderline-risk patients and when considering initiation of HMAs, given that treatment decisions have traditionally been guided by IPSS and/or IPSS-R data—areas where IPSS-M may offer more precise guidance. 

Overall, current trends in MDS classification and prognosis emphasize a shift away from morphological criteria, particularly the percentage of bone marrow blasts, toward an increased focus on genetic factors. At the same time, the new classification of myelodysplastic syndromes and the new IPSS-M prognostic score draw attention to the pathobiology of myelodysplastic syndromes, moving away from classification and prognostic criteria that do not differentiate between true biological progression of the disease and progression induced by external factors such as age and comorbidities. This evolution holds significant promise for patient care, as better-defined genetic subtypes could lead to the development of targeted therapies.

## Figures and Tables

**Table 1:** Comparison of 2016 vs. 2022 WHO Classifications of Myelodysplastic Neoplasms.

Feature	2016 WHO Classification	2022 WHO Classification
**Terminology**	Myelodysplastic syndromes (MDS)	Myelodysplastic neoplasms (Emphasizing neoplastic nature)
**Classification Basis**	Primarily **morphology** with some molecular integration	Greater emphasis on **molecular and genetic findings**
**MDS with Single Lineage Dysplasia (MDS-SLD)**	Defined by dysplasia in **one** hematopoietic lineage	**Removed**; now integrated into MDS-5q and other categories
**MDS with Ring Sideroblasts (MDS-RS)**	Required **≥15%** ring sideroblasts (or ≥5% if SF3B1 mutated)	Now part of **MDS with mutated *SF3B1***, emphasizing genetic driver
**MDS with Multilineage Dysplasia (MDS-MLD)**	Dysplasia in **≥2** lineages without excess blasts	Retained but with **greater molecular characterization**
**MDS with Excess Blasts (MDS-EB)**	MDS-EB1 (5–9% blasts) and MDS-EB2 (10–19% blasts)	Reclassified as **MDS with increased blasts (MDS-IB1 & MDS-IB2)**
**MDS with Isolated del(5q)**	Defined by isolated **5q deletion**, typically with low blasts	Renamed **MDS-5q**; ***TP53* mutations emphasized** as high-risk
**Prognostic Markers**	Relied on **IPSS-R** (cytogenetics, blasts, cytopenias)	Integrates **molecular risk factors** (e.g., *TP53*, *ASXL1*, *RUNX1* mutations)
**MDS/MPN Overlap Syndromes**	Included MDS/MPN, RARS-T, CMML, aCML	**Now distinct from MDS**, classified as **MDS/MPN neoplasms**
**TP53 Abnormalities**	Not separately classified	**New category: MDS with biallelic *TP53* inactivation** (high-risk)
**Genetic Integration**	Molecular findings considered but not central	**Key driver mutations (*SF3B1*, *TP53*, *ASXL1*, *RUNX1*, etc.) incorporated into classification**

Abbreviations: MDS-5q, Myelodysplastic Syndromes with isolated del(5q); MDS/MPN, Myelodysplastic Syndromes/Myelodysplastic Neoplasms; RARS-T, Refractory Anemia with Ring Sideroblasts associated with Thrombocytosis; CMML, Chronic Myelomonocytic Leukemia; aCML, Atypical Chronic Myeloid Leukemia, BCR-ABL1-negative.

**Table 2:** Comparison of WHO 2022 and ICC 2022 Classifications of Myelodysplastic Neoplasms.

Feature	WHO 2022 Classification	ICC 2022 Classification
**Terminology**	Myelodysplastic neoplasms (MDS); replaces ‘syndromes’ to emphasize **neoplastic nature**	Myelodysplastic neoplasms (MDS) terminology used; harmonized with modern nomenclature
**Overall structure**	Two broad groups: **genetically** defined MDS and **morphologically** defined MDS	Genetically informed framework; adds **transitional ‘MDS/AML’** category for 10–19% blasts
**Genetically defined entities**	MDS with mutated ***SF3B1***; MDS with biallelic ***TP53*** inactivation; MDS with isolated **del(5q)**	MDS with mutated ***SF3B1***; MDS with isolated **del(5q)**
**Morphological defined entities**	MDS with **increased blasts** (MDS-IB1: 5–9%; MDS-IB2: 10–19%); recognizes **hypoplastic** MDS and MDS with **marrow fibrosis** as distinct entities	Uses **‘MDS/AML’** to capture 10–19% blasts; otherwise mirrors morphologic categories without EB-2 label
**Blast threshold and AML definition**	Certain **AML-defining genetic alterations** allow AML diagnosis **irrespective of blast %**; otherwise blast % guides MDS subtyping	**Genetically defined AML entities** can be diagnosed with **≥10% blasts**; ‘MDS/AML’ names 10–19% blasts without AML-defining genetics
**Role of genetics vs. morphology**	Greater emphasis on **molecular/cytogenetic findings** alongside morphology	Similar shift toward molecular drivers; places naming **emphasis on proximity to AML** for 10–19% blasts
**Implications for management**	Subtyping (e.g., bi-TP53, SF3B1) **refines prognosis** and may influence **therapy selection**	Labeling of **‘MDS/AML’** highlights patients near **AML biology**, informing **intensity** and **timing of therapy**

Abbreviations: MDS, Myelodysplastic Neoplasms; AML, Acute Myeloid Leukemia.

**Table 3:** Comparison of IPSS, IPSS-R, and IPSS-M for Myelodysplastic Neoplasms.

Feature	IPSS (1997) [21]	IPSS-R (2012) [7]	IPSS-M (2022) [26]
**Main Basis of Classification**	Cytogenetics, blast percentage, cytopenias	More detailed cytogenetics, refined cytopenia & blast thresholds	Adds **molecular mutations** to improve risk stratification
**Risk Groups**	4 (Low, Intermediate-1, Intermediate-2, High)	5 (Very Low, Low, Intermediate, High, Very High)	6 (Very Low, Low, Moderate Low, Moderate High, High, Very High)
**Cytogenetic Risk**	Broad groups (Good, Intermediate, Poor)	Expanded with 5 groups (Very Good, Good, Intermediate, Poor, Very Poor)	Same as IPSS-R but integrates **molecular data**
**Cytopenia Assessment**	Counts cytopenias but not severity	Weighs severity of anemia, neutropenia, and thrombocytopenia	Same as IPSS-R but **adjusted for molecular findings**
**Blast Count in Bone Marrow**	<5% (low), 5–10% (intermediate), >10% (high)	More refined blast percentage cutoffs	Same as IPSS-R but with **molecular influence on risk**
**Molecular Mutations**	**Not included**	**Not included** (but molecular markers were being studied)	**Includes key mutations:** *TP53*, *ASXL1*, *RUNX1*, *SF3B1*, *DNMT3A*, etc.
**Prognostic Accuracy**	Limited predictive value in molecular era	Improved cytogenetic risk but still **lacks molecular data**	**Most accurate**, accounts for high-risk mutations & *TP53* biallelic alterations
**Applicability**	Used historically, but now outdated	Still widely used but less predictive without molecular testing	**Current gold standard for prognosis** when molecular data is available

## Data Availability

No new data were created or analyzed in this study.

## Abbreviations

The following abbreviations are used in this manuscript:

MDSs	Myelodysplastic Syndromes/Neoplasms
AML	Acute Myeloid Leukemia
FAB	French–American–British Classification of MDS
RARSs	Refractory Anemia with Ring Sideroblasts
RA	Refractory Anemia
RAEBs	Refractory Anemia with Excess Blasts
RAEB-T	Refractory Anemia with Excess Blasts in Transformation
CMML	Chronic Myelomonocytic Leukemia
WHO	World Health Organization
MDS-SLD	Myelodysplastic Syndromes with Single Lineage Dysplasia
MDS-5q	Myelodysplastic Syndromes with isolated del(5q)
MDS-RS	Myelodysplastic Syndromes with Ring Sideroblasts
MDS-MLD	Myelodysplastic Syndromes with Multiple Lineage Dysplasia
MDS-EB	Myelodysplastic Syndromes with Excess Blasts
MDS-IB	Myelodysplastic Syndromes with Increased Blasts
RARS-T	Refractory Anemia with Ring Sideroblasts associated with Thrombocytosis
aCML	Atypical Chronic Myeloid Leukemia, BCR-ABL1-negative
IPSS	International Prognostic Scoring System
IPSS-R	Revised International Prognostic Scoring System
WPSS	WHO Prognostic Scoring System
IPSS-M	Molecular International Prognostic Scoring System
MDS-biTP53	Myelodysplastic Syndromes with biallelic TP53 inactivation
HSCT	Hematopoietic Stem Cell Transplant
HMAs	Hypomethylating Agents
NGS	Next-Generation Sequencing
